# G-DOC *Plus* – an integrative bioinformatics platform for precision medicine

**DOI:** 10.1186/s12859-016-1010-0

**Published:** 2016-04-30

**Authors:** Krithika Bhuvaneshwar, Anas Belouali, Varun Singh, Robert M. Johnson, Lei Song, Adil Alaoui, Michael A. Harris, Robert Clarke, Louis M. Weiner, Yuriy Gusev, Subha Madhavan

**Affiliations:** Innovation Center for Biomedical Informatics, Georgetown University Medical Center, Washington, DC USA; Department of Oncology, Lombardi Comprehensive Cancer Center, Georgetown University, Washington, DC USA

**Keywords:** Bioinformatics, Translational research, Precision medicine, Cloud computing, Variant analysis, Next generation sequencing, Outcomes research, Genotype-phenotype integration

## Abstract

**Background:**

G-DOC *Plus* is a data integration and bioinformatics platform that uses cloud computing and other advanced computational tools to handle a variety of biomedical BIG DATA including gene expression arrays, NGS and medical images so that they can be analyzed in the full context of other omics and clinical information.

**Results:**

G-DOC *Plus* currently holds data from over 10,000 patients selected from private and public resources including Gene Expression Omnibus (GEO), The Cancer Genome Atlas (TCGA) and the recently added datasets from REpository for Molecular BRAin Neoplasia DaTa (REMBRANDT), caArray studies of lung and colon cancer, ImmPort and the 1000 genomes data sets. The system allows researchers to explore clinical-omic data one sample at a time, as a cohort of samples; or at the level of population, providing the user with a comprehensive view of the data.

G-DOC *Plus* tools have been leveraged in cancer and non-cancer studies for hypothesis generation and validation; biomarker discovery and multi-omics analysis, to explore somatic mutations and cancer MRI images; as well as for training and graduate education in bioinformatics, data and computational sciences. Several of these use cases are described in this paper to demonstrate its multifaceted usability.

**Conclusion:**

G-DOC *Plus* can be used to support a variety of user groups in multiple domains to enable hypothesis generation for precision medicine research. The long-term vision of G-DOC *Plus* is to extend this translational bioinformatics platform to stay current with emerging omics technologies and analysis methods to continue supporting novel hypothesis generation, analysis and validation for integrative biomedical research. By integrating several aspects of the disease and exposing various data elements, such as outpatient lab workup, pathology, radiology, current treatments, molecular signatures and expected outcomes over a web interface, G-DOC *Plus* will continue to strengthen precision medicine research. G-DOC *Plus* is available at: https://gdoc.georgetown.edu.

**Electronic supplementary material:**

The online version of this article (doi:10.1186/s12859-016-1010-0) contains supplementary material, which is available to authorized users.

## Background

The advent of the microarray technology in the year 2000 has paved the way for advanced translational research methods that have helped biologists analyze large group of genes as opposed to looking at one gene at a time. These methods have also been extended to other molecular markers such as microRNA, proteins, metabolites and DNA copy number data. Our flagship web platform, the Georgetown Database of Cancer (G-DOC) [1] was developed and deployed in April 2011 to enable the practice of an integrative translational and systems-based approach to research and medicine in cancer. G-DOC is a feature-rich shareable research infrastructure that allows physician scientists and translational researchers to mine and analyze a variety of “omics” data in the context of consistently defined clinical outcomes data for cancer patients.

This paradigm shift of looking at a group of markers has driven the development of next generation sequencing (NGS) platforms to analyze biological samples. The popularity of NGS grew exponentially since 2007 when faster, more accurate and affordable sequencing throughput became a reality [2, 3]. Since then, the size and complexity of genomic data has increased many fold, making its analysis, management and integration increasingly challenging [3].

Scientists today are using not only a combination of clinical, NGS and omics data for analysis, but also medical and digital images for validation of analysis results. Currently, numerous tools and software exist that specialize in the handling and processing of one or two “omics” data types, or only NGS data. Many of these systems require a bioinformatician to help with analysis. To drive hypothesis generation and validation of molecular markers for biologists and researchers, it would be convenient to have a “one-stop” system that can handle all of these data types, including NGS and medical images, in one location without having to switch to other tools or resources for analysis. For this purpose, we expanded the G-DOC system to support NGS and medical images. Moreover, the success of G-DOC in the cancer realm has helped us realize the importance of such systems in the non-cancer world for complex diseases including Alzheimer’s, and Duchene Muscular Dystrophy (DMD).

With the goal of improving overall health outcomes through genomics research, we present G-DOC *Plus*, a web-based bioinformatics platform that enables the integrative analysis of multiple data types to understand mechanisms of cancer and non-cancer diseases at a systems level for systematic conduct of research in precision medicine. G-DOC *Plus* currently holds data from over 10,000 patients selected from private and public resources including Gene Expression Omnibus (GEO), The Cancer Genome Atlas (TCGA) and the recently added datasets from REpository for Molecular BRAin Neoplasia DaTa (REMBRANDT), caArray studies of lung and colon cancer and the 1000 genomes data sets. G-DOC *Plus* allows researchers to explore clinical-omic data one sample at a time, as a cohort of samples; or at the level of population, providing the user with a comprehensive view of the data.

## Implementation

### Data

All data in G-DOC *Plus* are organized as “studies” on topics such as breast cancer, wound healing, or the 1000 Genomes project. Each study may contain de-identified clinical and biospecimen data, including mRNA and miRNA expression, copy number variation, metabolite mass spectrometry data; whole genome sequencing (WGS) data and medical images, currently adding to over 10000 patient and cell line datasets.

The data collection includes WGS data from the 1000 Genomes Project [4] and Complete Genomics [5]; multi-omics data from the NCI-60 data collection; numerous breast, GI, and pediatric cancer studies; and non-cancer studies including Duchenne muscular dystrophy (DMD) and Alzheimer’s disease from public resources including Gene expression Omnimus (GEO) [6] and TCGA [7]. In March 2015, data from REpository for Molecular BRAin Neoplasia DaTa (REMBRANDT) portal [8] (http://rembrandt.nci.nih.gov), and two studies from Ca Array [9] were migrated to G-DOC *Plus*. Most recently, two Infectious Disease (ID) studies were added to our data collection, one of which is from the Immunology Database and Analysis Portal (ImmPort) data collection [10].

**Fig. 1:**
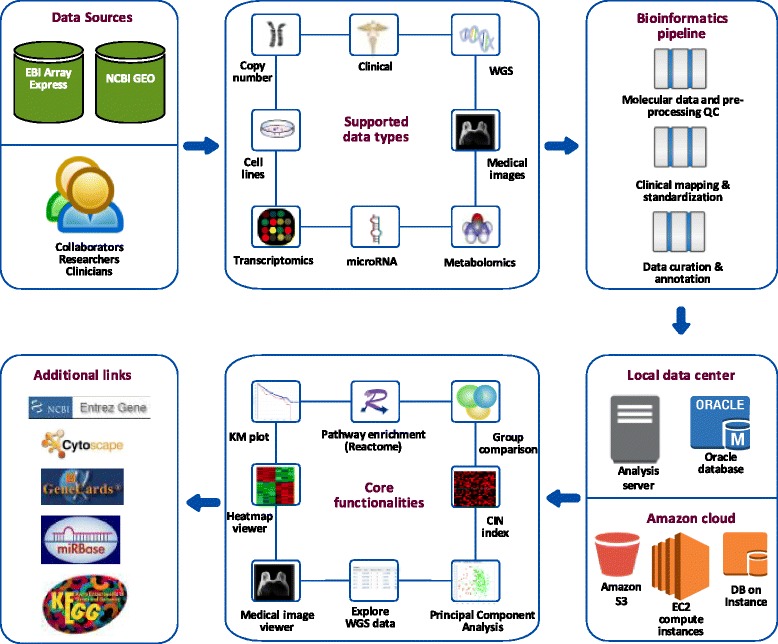
Summary of G-DOC *Plus* data management and analysis features

A summary of G-DOC *Plus* data management and analysis features is shown in Fig. 1, and a summary of currently available data collection can be seen on the front page of the software system at https://gdoc.georgetown.edu, also shown in Additional file 1.

### Tools and features

G-DOC *Plus* has three inter-connected entry points for the user based on their interests: (1) Precision Medicine workflow that allows researchers to explore data one sample at a time, (2) Translational Research workflow allows exploring data as a sub-cohort of samples and (3) Population Genetics workflow allows uses to look a population as a whole. This provides users with a comprehensive view of the data and facilitates hypothesis generation for basic, clinical, and translational researchers.

**Fig. 2:**
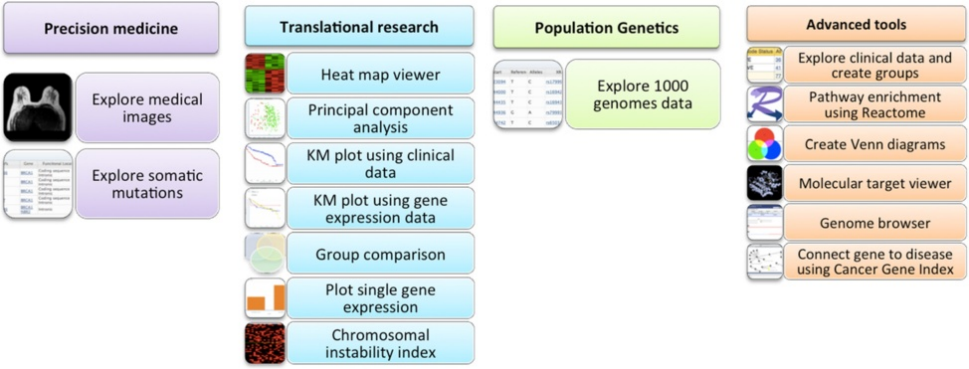
Summary of G-DOC *Plus* tools under each workflow

G-DOC *Plus* has a broad collection of tools that are summarized in the form of a schematic diagram in Fig. 2. It includes differential expression analysis, heat maps and hierarchical clustering, principal component analysis (PCA), survival analysis (Kaplan-Meier), tools to view copy number instability, interaction networks in Cytoscape, molecular targets; and a genome browser. It also includes a number of recently added tools including pathway enrichment (using Reactome database [11]) and tools to explore germline and somatic mutations and medical MRI images.

### System architecture

G-DOC *Plus* uses an in-house architectural framework that provides over 1500 biomedical research users (as of March 2016) with a comprehensive set of analysis routines and visualizations for a rich user experience through a web interface. The data and meta-data are stored in an Oracle database that allows for access control. The G-DOC *Plus* application is written in Groovy & Grails, an open source application framework that runs on the Java Virtual Machine (JVM) that is built on top of libraries such as Spring and Hibernate that meet high industry standards. This provides for a highly structured web application framework and allows for rapid development of modules. Adobe Flex and Javascript are used to provide interactive data visualizations. The system uses JBossMQ to communicate asynchronously with an analysis server on which analysis routines (such as classification, hierarchical clustering, etc.) are run using R/Bioconductor packages [12].

This architecture has since been expanded in G-DOC *Plus* to allow storage and analysis of NGS data and medical images by using EC2 instances [13] in the Amazon cloud computing environment [14]. These compute intensive instances offer the ability to rapidly scale in the face of a deluge of data at a very low maintenance cost.

Genomic data by its very nature is very complex and subject to changes due to rapidly changing knowledge and technologies in the field of genomics. Traditional SQL databases require highly structured and well-defined data and are not flexible enough to handle such rapid changes. For large hierarchical datasets such as NGS, transactions on relational databases can be very expensive [15]. NoSQL [16] databases such as MongoDB [17] are best suited for such applications. They lack the rigorous consistencies of SQL in favor of rapid development in the face of changing data. According to the Consistency, Availability, and Partition tolerance (CAP) theorem [18], these NoSQL databases tend to focus more on availability and partition tolerance than consistency (as opposed to SQL databases which focus more on consistency). This makes them ideal for housing, indexing and retrieving big data.

We utilized MongoDB to store the variant data from sequencing studies because of its inherent scalability and high performance on very large data sets. This database is located in an EC2 server instance on the Amazon cloud. Each NGS dataset is stored as a “collection” [17]. The data are indexed so that data retrieval is fast and efficient. Every record in the database is a unique “document” [19] in the form of BSON (binary JSON) objects [20], and holds variant data and meta-data in the form of key-value pairs. This JSON based data model allows for adding new attributes such as annotation attributes without changing the underlying database design. This allows for a very flexible implementation that can be changed as new annotation types emerge. The G-DOC *Plus* web application layer executes queries against the mongoDB database using web services that are exposed by a Python based Django [21] application. This web service crafts and parses the query, and extracts the results for the end user.

The medical MRI images are in the form of standard Digital Imaging and Communications in Medicine (DICOM) objects [22]. In G-DOC *Plus*, the images are stored in a DICOM Clinical Data Manager “Dcm4chee” system. This system sits on a JBoss web application that is hosted on an EC2 server instance on the Amazon cloud. The meta- data of the images are stored in a MySQL database, also located on the EC2 server. The images along with its meta-data are archived comprehensively within the Dcm4chee system to allow for easy extraction and presentation in G-DOC *Plus*. The EC2 server also contains Oviyam [23], a cross platform DICOM viewer that was integrated to enable web based access to the medical images. Both Dcm4chee and Oviyam are open source tools and they allow for exploration of medical images and associated meta-data. The medical images are also linked to the phenotypic data of the patient, which allows filtering and selection of images for a specific patient based on their clinical data.

**Fig. 3:**
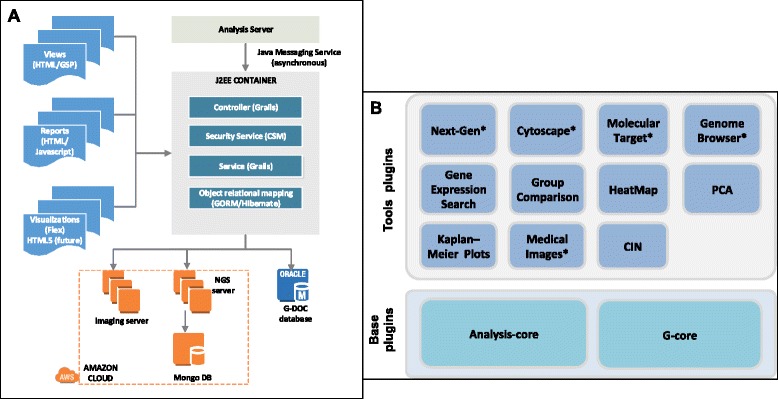
**a** General system architecture of G-DOC *Plus*. **b** Summary of plugins in G-DOC *Plus*. The plugins marked in * indicate open source plugins that were customized for G-DOC *Plus*

Figure 3a shows an overview of the architecture, including how the system interacts with the cloud instances to retrieve NGS and medical images. As of March 2016, the system hosts 82 GB of clinical and other omics data, 152 GB of processed NGS data, 56 GB of medical images and 720 MB of meta-data.

### The G-DOC *Plus* ecosystem

The G-DOC *Plus* ecosystem is a generalized concept that was realized for easy replication of the G-DOC *Plus* tools for similar applications. It consists of many independent “plugins” built on top of two base plugins to form a comprehensive ecosystem. A plugin is an independent lightweight “mini” grails project that cannot be run on its own on a web server. Figure 3b shows the plugins used in G-DOC *Plus*.

G-core is a ‘core’ grails plugin created and installed into a skeleton grails application to provide a uniform database schema, common security framework that includes authentication, authorization, registration and basic features of a portal (exploring clinical data, saving patient lists, workflows). This is the biggest and most important plugin, and in principle the G-DOC application can be run with only the G-core plugin without any other plugin. The analysis-core plugin is another ‘core’ plugin developed to create communication between the analysis server and any type of analysis that requires computation. All plugins have a dependency on g-core plugin and those that call the analysis server are also dependent on the analysis-core plugin. The primary analysis features are built as plugins on top of these two base plugins, perform specific tasks for data management or analysis, and are independent of each other.

Such an ecosystem allows for easy creation of new plugins based on new analyses requirements, and independent installation into any application that already has the ‘core’ plugins installed. This module-based system hence gives us the flexibility to pick the necessary plugins to be able to build a G-DOC like web portal for another collaborator/application.

All source code for G-DOC *Plus* is available in Github [24]. It is currently a private repository and can be freely shared with academic collaborators or developers after establishing a Memorandum of Understanding (MOU) with Georgetown University.

## Results and discussion

As described previously, The Georgetown Database of Cancer (G-DOC) was developed and deployed 5 years ago; at that time, it contained only cancer datasets and tools to handle multi-omics data. The goal was to enable a translational and systems-based approach to research and medicine in cancer. The tools available in the system at the time are now part of the “Translational Research” module in G-DOC *Plus*.

The success of G-DOC in the cancer realm helped us realize the importance of such systems in the non-cancer world for complex diseases including Alzheimer’s, and Duchene Muscular Dystrophy (DMD). Scientists today are using not only clinical and targeted omics data, but also Next Generation Sequencing (NGS) data, medical and digital images for a variety of analysis. To drive hypothesis generation and validation of molecular markers for biologists and researchers, it would be convenient to have a “one-stop” system that can handle various data types, including NGS and medical images, in one location without having to switch to other tools or resources for analysis. For these reasons, we significantly expanded the G-DOC system to include non-cancer datasets and newer data types (hence the name G-DOC “*Plus*”).

Apart from our cancer datasets collection, G-DOC *Plus* currently includes data on various non-cancer diseases including Dementia, DMD and Wound healing. We are continuing to enrich this collection and most recently added two Infectious disease datasets.

In addition to the Translational research module, G-DOC *Plus* includes two new modules –Precision medicine and Population genetics modules. To enable use cases in these additional modules, new tools (“Variant Search”, “Explore Medical Images”, “Phenotype Search”), and datasets were added to the system (NGS data from The 1000 Genomes project and Complete Genomics Breast Cancer dataset; and TCIA MRI imaging data).

The overall user interface was redesigned to provide better end user experience in G-DOC *Plus*. Also, the web interface of the menu option: “Explore clinical data and create groups” (the clinical data exploratory tool) was upgraded with a new user-friendly interface that offered total counts for each combination of variables selected. In addition, the backend architecture was re-designed into a modular structure (Grails plugin architecture) allowing for a flexible and extendible framework for new analysis modules to be easily added to the platform. Another enhancement to the architecture allows for whole genome sequencing (WGS) data and the MRI images to be stored on a cloud infrastructure.

We have continued to improve our cancer data collection by adding new datasets including three large data collections transitioned from NCI to G-DOC *Plus*: the REMBRANDT brain cancer dataset, Colon cancer and Lung Cancer datasets (both from NCI caArray platform) as well as NCI-60 cell line collection and Pediatric cancer datasets.

A comprehensive table summarizing the differences between G-DOC *Plus* and G-DOC has been provided in Additional file 2.

G-DOC *Plus* can be used to support a variety of user groups in multiple domains to enable hypothesis generation for precision medicine research. The tools in G-DOC *Plus* have been leveraged to support numerous case studies, some of which are described below to demonstrate its multifaceted usability.

### Case study 1: drug metabolizer status search

Common variants in the CYP family cause modified drug function, and these variants are known to differ greatly by ethnicity. Patients are classified into groups referred to as “drug metabolizer status” or “phenotype” based on their modified drug function. These groups are: (a) Ultra Rapid Metabolizers (UM) - have 5–6 copies of the variant (due to gene amplification) that have a high response to the drug (b) Extensive Metabolizers (EM) – Have two copies of the variant and have a normal response to the drug (c) Intermediate Metabolizers (IM) - Have one copy of variant and have a medium response to the drug and (d) Poor Metabolizers (PM) – have both copies of the variant deleted and have low response or adverse effect to the drug [25].

**Fig. 4:**
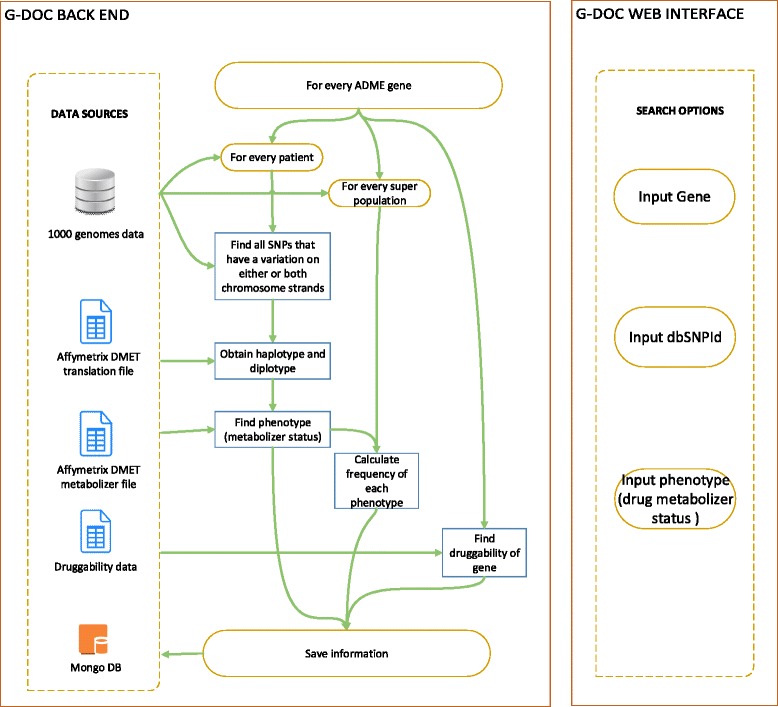
The workflow used to extract information for the 1000 genomes dataset in G-DOC *Plus*. The pre-processed data was about 132 GB in size and stored in Mongo DB on the Amazon cloud for quick and efficient data retrieval

This use case involved understanding racial/ethnic distribution of dysfunctional *genotypes* and genotype-derived *phenotypes* (i.e., haplotypes) for absorption, distribution, metabolism and excretion (ADME) related genes. To enable this, we integrated public genome data from the 1000 genomes project [4] with data from the Affymetrix Drug Metabolizing Enzymes and Transporter (DMET) console [26] and calculated the haplotypes and phenotype (drug metabolizer status) for the ADME genes. Out of ~2000 ADME genes, metabolizer status information was available for 20 genes. Variations were found in 19 of the 20 genes for at-least one person in the dataset. The frequency of the drug metabolizer status was then calculated for each of the five super populations in the dataset for each gene. The druggability of the gene was obtained from Sophic Alliance’s Druggable Genome Database [27]. The steps used in the data processing of this workflow for G-DOC *Plus* is shown in Fig. 4. This pharmacogenomics (PGx) workflow allows the user to perform searches and filter results using gene, dbSNP id [28] or drug metabolizer status.

#### Example query: *How common is the “poor metabolizer” phenotype in CYP2C19 in the 1000 genomes dataset?*

CYP2C19 is known to be involved in clopidogrel (Plavix) metabolism, and the activity of the drug depends on the active metabolite. Users can query the G-DOC *Plus* interface to see the frequency of PMs and UMs in the 1000 genomes dataset, which has been summarized in Table 1. We saw that most of the poor and intermediate metabolizers in this dataset were East Asians, which is consistent with literature [29–31]. On the other hand, majority of the ultra rapid and extreme metabolizers were Europeans and Africans. A screen shot of this on G-DOC *Plus* is shown in Additional file 3.

**Table 1:** Summary of modified drug function for CYP2C19 by ethnicity in the 1000 genomes dataset exported from G-DOC *Plus* (rounded to the nearest decimal)

Phenotype (Drug metabolizer status)	Ad-mixed American (AMR) %	African (AFR) %	East Asian (ASN) %	European (EUR) %	South Asian (SAN) %
UM (*17/*17)^a^	2	6	0	5	0
UM or EM (*1/*17)	16	23	2	29	0
EM	55	35	31	38	0
IM	23	22	54	19	0
PM (*2/*2, *2/*3, *3/*3)	**1**	**3**	**11**	**1**	**0**
Not PM	3	9	2	8	0
IM or PM	0	1	0	0	0
Unknown/NA	0	1	0	0	0
TOTAL %	100	100	100	100	0

The table shows that about 11 % of the East Asians were PMs, ~ 3 % Africans, and ~ 1 % Ad mixed Americans were PMs (row shown in bold font). We can see the opposite trend for UM where ~ 5 % Europeans, ~ 6 % of Africans, ~ 2 % of Ad-mixed Americans were UMs, none of the East Asians were UMs (row marked with ^a^)

Such a resource can help a researcher query the interface to assess if a given ADME gene(s) is polymorphic or not; or extract the racial/ethnic distribution of metabolizer status for a gene of interest. This information can be used (a) to assess whether an individual with a SNP of interest will have efficacy to a drug or not (b) to help understand SNP population frequencies for specific drug targets in new drug applications, which can help inform broad applicability of a drug to different populations. A new drug’s sponsor can evaluate all relevant target variants in various subpopulations and (c) to adjust drug dosage, as UMs may need a smaller dose to get required efficacy compared to EMs; while an alternative drug treatment may be recommended for PMs [25].

In the future, we plan to extend this workflow to all genes. This unique new tool created using public data can be used to create genomic profiles for precision medicine research with a broad range of applications in pharmacogenomics.

### Case study 2: exploratory analysis of germline and somatic variations

Users can explore somatic and germline variants in cancer samples using the G-DOC system. The “variant search” tool allows users to explore and filter mutations based on genes, chromosomes, functional location (*coding sequence, intronic, downstream of gene, upstream of gene, 5′ UTR, 3′ UTR, non-coding UTR, intergenic*), and exonic function (*Loss of stop, Premature stop, Insertion, Deletion, Frameshift, Substitution, Possible splice variant, Possible 5′ splice variant*).

The inactivation of tumor suppressor genes could be caused by accumulation of mutations or, loss of heterozygosity (LOH), causing progression of cancer [32, 33]; one such location is Chromosome 8, and abnormalities in this chromosome have been reported in breast, colon and other cancer [34, 35]. This led us to the ***Example query: which genes are affected by novel deletions in chromosome 8 with potential impact on protein function, and what major pathways might these genes be involved in? Is there a networked relationship between one or more of these impacted genes?***

The dataset used for this query was WGS data on breast cancer cell lines from Complete Genomics [36]. This data was processed and stored in Mongo DB on the Amazon cloud.

**Fig. 5:**
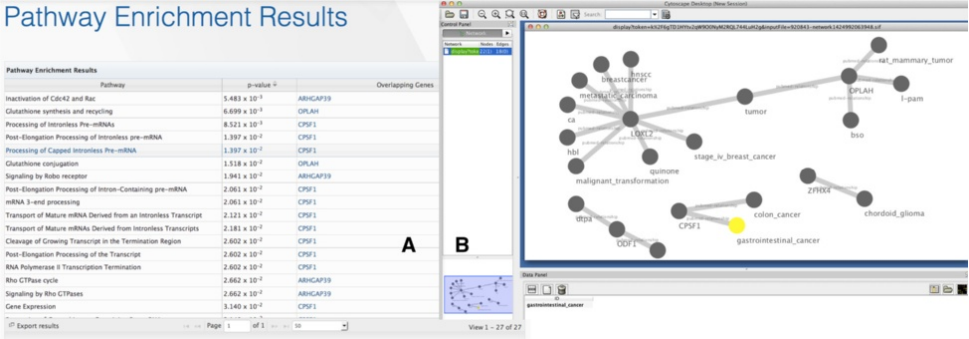
**a** and **b** Pathway enrichment and gene network in G-DOC *Plus*

To find the answer to this query, we chose the following settings on the G-DOC *Plus* interface: *Chromosome: 8; Functional Location: coding sequences; Exonic function: Non-synonymous, Deletion; and Variant Inclusion: Novel*; which resulted in a list of seven variants from seven different genes (Additional file 4 shows a screen shot of the G-DOC *Plus* web page with these settings). We saved the gene list from this result and performed pathway enrichment analysis in G-DOC *Plus.* Many of the pathways enriched were related to processing of pre-mRNA and mature mRNA, which are known to potentially alter gene expression and in-turn turn tumor suppressor genes off [37]. The top pathway “Inactivation of CD42 and RAC” indicated that when such RNA processing pathways are turned negative, it causes other tumor pathways to go out of control. The saved gene list was also used to create a Cytoscape [38] network from within G-DOC *Plus*. This cytoscape network showed annotation with diseases and molecules based on Cancer Gene Index [39] (Fig. 5a and b).

This case study shown on cell line data, which represents individual tumors can be applied on other patient datasets to allow in-depth analysis of germline mutations for individual patients based on NGS data. The variant search tool also allows exploration of somatic variants (variants present in tumor, but not in matched normal sample).

### Case study 3: perform multi-omics analysis

G-DOC *Plus* has recently enabled the detection of prognostic markers using multi-omics data for relapse in colorectal cancer samples [40]. To demonstrate this functionality in G-DOC *Plus*, ***we present an example query that compared patients with Astrocytoma (low grade glioma) with those with Gliobastoma (GBM, high grade glioma) in the NCI REMBRANDT study.***

**Fig. 6:**
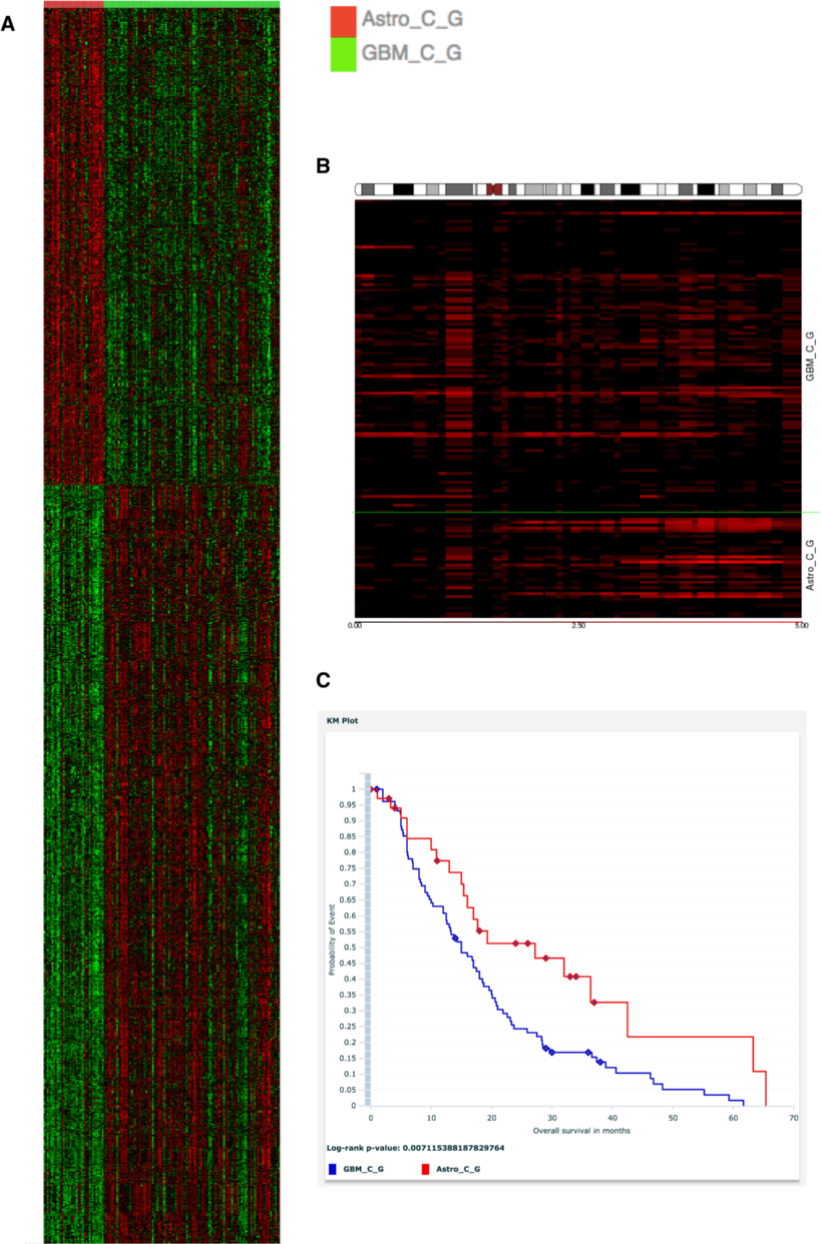
**a** Heat map comparing Astrocytoma and GBM patients. Over-expression of genes in the heat map is represented in red color, and under-expression is shown in green color. **b** Chromosome instability in chromosome 8. Here, black color indicates normal DNA copy number (i.e. no instability); and the red color indicates instability - higher the instability, the brighter the red color on the heatmap. **c** Kaplan Meier survival plot between Astrocytoma (*red line*) and Glioblastoma patients (*blue line*)

Only patients that had gene expression, copy number and clinical data available were considered for this case study. Additional file 5 shows a screen shot of this cohort selection within G-DOC *Plus*. We first applied a T-test with FDR correction [41] to find 1692 differentially expressed genes (DEGs) with p-value cut off of 0.0001 and fold change cut off of 1.5. The most down-regulated gene RHOF was six fold under-expressed in the GBM group compared to the Astrocytoma group. This gene is known to be down regulated in GBM patients through the over expression of their activators [42]. Similar changes in expression in another under-expressed gene MLNR (5.9 fold down in GBM group) were found in low-grade gliomas of Chinese patients [43]. Among the other DEGs, were CYP4A11, TXN, MYLK, previously shown to be to be over expressed, and COL9A3 previously shown to be under-expressed in high-grade gliomas respectively [43, 44]. A screen shot of this comparative analysis is shown in Additional file 6, and a heatmap of DEG expression values is shown in Fig. 6a.

We also examined DNA copy number data for these patients using the chromosomal instability tool in G-DOC *Plus*. From comparison of copy number data between the two glioma types (Additional file 7), we found a higher level of chromosomal instability in the Astrocytoma group in chromosome 8q arm (indicated by the bright red colors). Aberrations in the 8q arm in Astrocytoma patients are known in literature [45–47] (Fig. 6b). The list of differentially changed cytobands (Additional file 7) also showed higher instability in GBM compared to Astrocytoma in the 7p and 10q regions. These 7p and 10q regions are known to be highly amplified in GBM patients [48–51]. Higher genomic instability is also synonymous with aggressive phenotype especially in cancer.

Finally, we looked at the overall survival of these patients using the Kaplan Meier survival plot (Fig. 6c) feature in G-DOC *Plus*, which showed the expected result that patients with Astrocytoma (low grade glioma) had better survival rates than those with GBM (high grade glioma) with a *p*-value of less than 0.05 from log rank test.

The CIN index algorithm [52] currently implemented in G-DOC *Plus* summarizes both gains and losses of copy number as an “instability” (referred to as “overall CIN index”). As an upcoming feature in G-DOC *Plus*, we plan to enable analysis of gains and losses of copy number separately, in addition to “overall CIN index”. This CIN module is available to the public in Bioconductor (http://bioconductor.org/packages/CINdex/).

This kind of platform can enable users to generate new hypotheses from existing data. There is often an added value in obtaining new insights into the etiology, diagnosis, treatment, and prevention of diseases from re-analyzing published datasets. G-DOC *Plus* can be used to perform such in silico meta-analysis of disparate studies. An example of such an in silico analysis done on three ER+ breast cancer studies in G-DOC *Plus* was done as part of the NCI In Silico Research Centers of Excellence Program [53].

### Case study 4: explore medical images in conjunction with clinical data

G-DOC *Plus* allows researchers and radiologists to explore medical images associated with clinical outcome using the Digital Imaging and Communications in Medicine (DICOM) format. We currently have a public breast cancer imaging study obtained from The Cancer Imaging Archive (TCIA) (cancerimagingarchive.net) [54] in the system to allow users to explore this functionality.

**Fig. 7:**
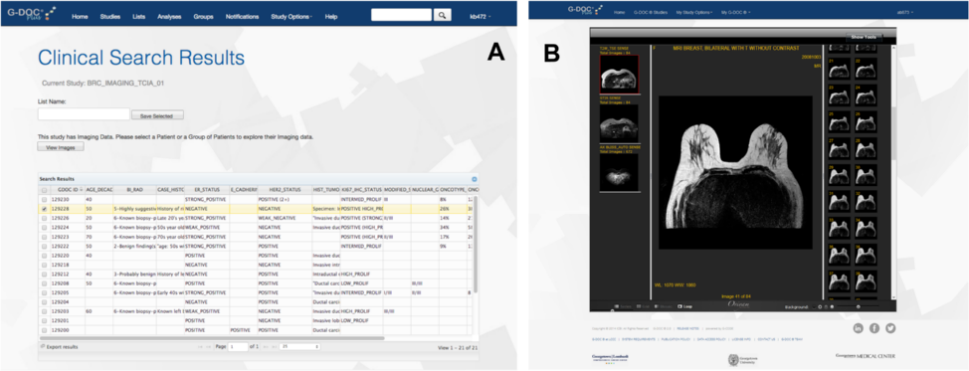
**a** Selection of medical images based on clinical data of patient(s). **b** Screen shot of the medical MRI imaging module in G-DOC *Plus*

This module allows investigators secure access to view aggregate data, perform simple data analysis, and run ad hoc queries across the data. Since the medical images on the cloud are linked to the clinical data of the patient in the Oracle database, it allows filtering and selection of images for a specific patient based on their clinical data. Users can explore view images of all performed procedures for all or any patient(s); or select a patient(s) based on specific clinical attributes in the study before viewing the imaging metadata (Fig. 7a). This can help correlate large data sets with radiology information and identify specific patterns in the data.

In the future, we plan to add more imaging data from many diseases including breast cancer and traumatic brain injury (TBI). We also envision additional capabilities to handle images that help visualize added chemicals (e.g. diagnostic dyes) within blood vessels, allow labeling of specific metabolites in real time, and vascularization of tumors. Such an imaging module will allow users to conduct a comprehensive serial analysis of integrated clinical, imaging, molecular, and genetic data from patient cohorts. Figure 7b shows a screen shot of this module.

### G-DOC *Plus* as a tool for in-silico confirmation of lab results

An increasing number of journals are asking biologists to confirm their findings using well-known public studies (such as TCGA, 1000 genomes) as part of validation of their results. Many biologists have approached our group at Georgetown-ICBI (Innovation Center for Biomedical Informatics) with results from their lab to seek our help in finding supporting evidence for confirmation of their lab findings in publicly available studies. We use a number of tools for this, including cBio portal [55], G-DOC and Oncomine [56]. We have worked with scientific collaborators in the breast and liver cancer areas and have been able to confirm lab findings using public studies in G-DOC as demonstrated in these publications [57–59].

G-DOC *Plus* has also been used by researchers for new hypothesis generation and for finding suitable datasets for their analysis as shown in these publications [60, 61].

### G-DOC *Plus* for education and the community

Since 2012, G-DOC and its newer version G-DOC *Plus* have been used as a hands-on tool to train scientists and teach graduate students a variety of concepts in molecular profiling data analysis in a context of translational bioinformatics [62].

G-DOC *Plus* hosts a number of well-known public studies where the raw data was downloaded, processed uniformly and then uploaded to our system, including TCGA Ovarian cancer dataset, the 1000 genomes dataset, the NCI-60 cell line collection, two studies from the CaArray collection, the REMBRANDT study, and one study from ImmPort. We have recently added five pediatric cancer studies to G-DOC *Plus*, which help researchers analyze individual molecular data types to identify biomarkers. Hosting such well-known studies allows for easy reproducibility of results. Such analyses enables users to better understand molecular changes that lead to disease or phenotype of interest.

G-DOC *Plus* has also been used as a resource for hosting public data. The clinical and multi-omics data from this publication [40] has been made available to the public through G-DOC *Plus* via the CRC_MADHAVAN_2013_01 study*,* and we hope to encourage owners of other private datasets in making their data public through our platform for wider access by the biomedical research community.

### Comparison of G-DOC *Plus* with similar tools

Three competing tools used for translational research include the cBio cancer genomics portal [55], TranSMART [63] and Oncomine [56]. The cBio cancer genomics portal is a free online tool that has processed data from many cancer genomics studies including the TCGA collection. TranSMART allows scientists to develop and refine research hypotheses by investigating correlations between phenotypic and omics data. Oncomine is another translational bioinformatics platform, but has limited functionality and access to data in its freely available version. We briefly summarized the differences between these tools in Table 2. A comprehensive comparison of these translational bioinformatics platforms including G-DOC has been summarized in these papers [64–66].

**Table 2:** Comparison of G-DOC *Plus* with other bioinformatics software platforms

	G-DOC *Plus*	TranSMART	CBio	Oncomine
Tools for Translational research	YES	YES	YES	YES
User friendly filtering of clinical data by user selected variables	YES	NO	NO	NO
Chromosomal instability index module that allows comparison of groups and plotting heatmaps at sample, chromosome, and cytoband level	YES	NO	NO	NO
Include tools such as Molecular Target Viewer to explore drug targets, Reactome for pathway analysis, and Cytoscape for network creation, integrated to allow end to end analysis	YES	YES	NO	NO
Tool to explore MRI medical images for precision medicine research	YES	NO	NO	NO
Variant search tool for precision medicine	YES	YES	NO	NO
Public studies available to the public for free	YES	Have a demo portal with limited access	YES	Limited free version
Tool to explore 1000 genomes dataset in the context of population genetics	YES	NO	NO	NO

## Conclusions

The data and tools in G-DOC *Plus* have enabled multi-modal inferences across cancer studies. We have leveraged this to support the detection of prognostic markers for relapse in colorectal cancer samples; to detect key metabolites related to disease severity; to identify progression and responsiveness to corticosteroid treatment in children with DMD; and to examine breast cancer MRI images for generating new research hypothesis.

In the future, we plan to enable users to upload their pre-processed data for multi-omics and NGS data analysis. We are planning to expand our data collection to add more next generation sequencing studies, medical imaging and non-cancer datasets. We also hope to add features including allowing users to see gains and losses in copy number, support for siRNA data, improved enrichment analysis and visualizations using newer technologies.

The long-term vision of G-DOC *Plus* is to extend this translational bioinformatics platform to stay current with emerging omics technologies and analysis methods to continue supporting novel hypothesis generation, analysis and validation for integrative biomedical research. By integrating several aspects of the disease and exposing various data elements, such as outpatient lab workup, pathology, radiology, current treatments, molecular signatures and expected outcomes over a web interface, G-DOC *Plus* will continue to strengthen precision medicine research.

## Availability and requirements

- **Project name:** G-DOC *Plus*
- **Project home page:**https://gdoc.georgetown.edu
- **Tutorials and demo video:**https://gdoc.georgetown.edu/tutorials
- **Access:** Any web browser, preferably Mozilla Firefox 3.5+ or Google Chrome
- **Web browser requirements:** Flash 10.0+, Java 1.6+ This requirement can be checked here: https://gdoc.georgetown.edu/gdoc/home/requirementCheck
- **Other information:** G-DOC *Plus* is freely available without restrictions to all users. Registration and acceptance of terms of use are required before first login.

## Additional files

Additional file 1: Front page of G-DOC *Plus* showing total number of studies, and samples in various disease types. (PNG 885 kb) Additional file 2: What’s new in G-DOC *Plus*? (DOCX 87 kb) Additional file 3: Screen shot of 1000 genomes use case with settings for poor metabolizers. (PNG 133 kb) Additional file 4: Screen shot of variant search use case with the settings. (PNG 265 kb) Additional file 5: Screen shot of G-DOC *Plus* showing clinical cohort creation. (PNG 424 kb) Additional file 6: T-test with FDR performed on gene expression data Astrocytoma and GBM patients. Screen shot of results on G-DOC *Plus* to show DEGs. (PNG 344 kb) Additional file 7: Rembrandt *T*-test with CIN cytobands. (PNG 344 kb)

## Abbreviations

ADME: absorption, distribution, metabolism and excretion
Chr: chromosome
DEGs: differentially expressed genes
DMD: Duchene muscular dystrophy
DMET: drug metabolizing enzymes and transporters
EM: extensive metabolizers
GEO: gene expression omnibus
HCC: hepatocellular carcinoma
ID: infectious disease
IM: intermediate
IPA: ingenuity pathway analysis
NGS: next generation sequencing
PM: poor metabolizers
REMBRANDT: REpository for Molecular BRAin Neoplasia DaTa
TCGA: the cancer genome atlas
UM: ultra rapid metabolizers (UM)
WGS: whole genome sequencing

## References

[1] Madhavan S, Gusev Y, Harris M, Tanenbaum DM, Gauba R, Bhuvaneshwar K (2011). G-DOC: a systems medicine platform for personalized oncology. Neoplasia.

[2] Editorial. Method of the Year. Nature methods. 2008;5:1. 10.1038/nmeth1153 18175409

[3] Schuster SC (2008). Next-generation sequencing transforms today’s biology. Nat Methods.

[4] The 1000 genomes data. [http://www.1000genomes.org/] Accessed 7 Oct 2015.

[5] Complete Genomics. [http://www.completegenomics.com] Accessed 9 Feb 2015.

[6] Barrett T, Wilhite SE, Ledoux P, Evangelista C, Kim IF, Tomashevsky M (2013). NCBI GEO: archive for functional genomics data sets--update. Nucleic Acids Res.

[7] TCGA Research Network. [http://cancergenome.nih.gov/] Accessed 9 Feb 2015.

[8] Madhavan S, Zenklusen JC, Kotliarov Y, Sahni H, Fine HA, Buetow K (2009). Rembrandt: helping personalized medicine become a reality through integrative translational research. Mol Cancer Res.

[9] Microarray data management system (caArray). [https://wiki.nci.nih.gov/display/caArray2/caArray] Accessed 23 Feb 2015.

[10] Bhattacharya S, Andorf S, Gomes L, Dunn P, Schaefer H, Pontius J (2014). ImmPort: disseminating data to the public for the future of immunology. Immunol Res.

[11] Vastrik I, D’Eustachio P, Schmidt E, Gopinath G, Croft D, de Bono B (2007). Reactome: a knowledge base of biologic pathways and processes. Genome Biol.

[12] Gentleman RC, Carey VJ, Bates DM, Bolstad B, Dettling M, Dudoit S (2004). Bioconductor: open software development for computational biology and bioinformatics. Genome Biol.

[13] Amazon EC2. [http://aws.amazon.com/ec2/] Accessed 9 Feb 2015.

[14] Amazon Web Services. [http://aws.amazon.com/] Accessed 26 Feb 2015.

[15] Can big data and SQL get along? [http://www.simba.com/blog/sql-access-non-relational-sources-proliferating/] Accessed 9 Feb 2015.

[16] NoSQL. [http://en.wikipedia.org/wiki/NoSQL] Accessed 9 Feb 2015.

[17] MongoDB. [http://www.mongodb.org/] Accessed 9 Feb 2015.

[18] CAP theorem. [http://en.wikipedia.org/wiki/CAP_theorem] Accessed 9 Feb 2015.

[19] Mongo DB Document Databases. [https://www.mongodb.com/document-databases] Accessed 7 Oct 2015.

[20] BSON. [http://bsonspec.org/] Accessed 23 Oct 2015.

[21] Django. [https://en.wikipedia.org/wiki/Django_(web_framework)] Accessed 23 Oct 2015.

[22] Digital Imaging and Communications in Medicine (DICOM). [http://dicom.nema.org/] Accessed 12 Mar 2015.

[23] Oviyam. [http://oviyam.raster.in/] Accessed 12 Mar 2015.

[24] Github. [https://github.com/] Accessed 9 Feb 2015.

[25] Cleveland heart lab CYP2C19. [http://www.clevelandheartlab.com/wp-content/uploads/2013/09/CYP2C19-Practitioner-OnePager-CHL-D022.pdf] Accessed: 9 Feb 2015.

[26] Affymetrix DMET console. [http://www.affymetrix.com/estore/browse/level_seven_software_products_only.jsp?productId=131559-1_1] Accessed.

[27] Griffith M, Griffith OL, Coffman AC (2013). DGIdb - Mining the druggable genome. Nature methods.

[28] Sherry ST, Ward MH, Kholodov M, Baker J, Phan L, Smigielski EM (2001). dbSNP: the NCBI database of genetic variation. Nucleic Acids Res.

[29] Dean L (2013). Clopidogrel therapy and CYP2C19 genotype. Medical genetics summaries.

[30] Shuldiner AR, O’Connell JR, Bliden KP, Gandhi A, Ryan K, Horenstein RB (2009). Association of cytochrome P450 2C19 genotype with the antiplatelet effect and clinical efficacy of clopidogrel therapy. JAMA.

[31] Desta Z, Zhao X, Shin JG, Flockhart DA (2002). Clinical significance of the cytochrome P450 2C19 genetic polymorphism. Clin Pharmacokinet.

[32] Karnik P, Paris M, Williams BR, Casey G, Crowe J, Chen P (1998). Two distinct tumor suppressor loci within chromosome 11p15 implicated in breast cancer progression and metastasis. Hum Mol Genet.

[33] Callahan R, Campbell G (1989). Mutations in human breast cancer: an overview. J Natl Cancer Inst.

[34] Dahiya R, Perinchery G, Deng G, Lee C (1998). Multiple sites of loss of heterozygosity on chromosome 8 in human breast cancer has differential correlation with clinical parameters. Int J Oncol.

[35] El Gammal AT, Bruchmann M, Zustin J, Isbarn H, Hellwinkel OJ, Kollermann J (2010). Chromosome 8p deletions and 8q gains are associated with tumor progression and poor prognosis in prostate cancer. Clin Cancer Res.

[36] Complete Genomics Breast Cancer Dataset. [http://www.completegenomics.com/public-data/cancer-data/] Accessed: 26 Feb 2015.

[37] Neel H, Gondran P, Weil D, Dautry F (1995). Regulation of pre-mRNA processing by src. Curr Biol.

[38] Shannon P, Markiel A, Ozier O, Baliga NS, Wang JT, Ramage D (2003). Cytoscape: a software environment for integrated models of biomolecular interaction networks. Genome Res.

[39] Cancer Gene Index. [https://wiki.nci.nih.gov/display/cageneindex/Cancer+Gene+Index+End+User+Documentation] Accessed: 26 Feb 2015.

[40] Madhavan S, Gusev Y, Natarajan TG, Song L, Bhuvaneshwar K, Gauba R (2013). Genome-wide multi-omics profiling of colorectal cancer identifies immune determinants strongly associated with relapse. Front Genet.

[41] Benjamini Y, Yekutieli D (2001). The control of the false discovery rate in multiple testing under dependency. Ann Statist.

[42] Fortin Ensign SP, Mathews IT, Symons MH, Berens ME, Tran NL (2013). Implications of Rho GTPase signaling in glioma cell invasion and tumor progression. Front Oncol.

[43] Li Y, Wang D, Wang L, Yu J, Du D, Chen Y (2013). Distinct genomic aberrations between low-grade and high-grade gliomas of Chinese patients. PLoS One.

[44] Rickman DS, Bobek MP, Misek DE, Kuick R, Blaivas M, Kurnit DM (2001). Distinctive molecular profiles of high-grade and low-grade gliomas based on oligonucleotide microarray analysis. Cancer Res.

[45] Duffau H (2013). Diffuse low-grade gliomas in adults.

[46] Sawyer JR, Thomas JR, Teo C (1995). Low-grade astrocytoma with a complex four-breakpoint inversion of chromosome 8 as the sole cytogenetic aberration. Cancer Genet Cytogenet.

[47] Felicella MM, Hagenkord JM, Kash SF, Powers MP, Berger MS, Perry A (2012). A common 8q (MYC) amplification detected in a multifocal anaplastic astrocytoma by SNP array karyotyping. Clin Neuropathol.

[48] Lopez-Gines C, Cerda-Nicolas M, Gil-Benso R, Pellin A, Lopez-Guerrero JA, Callaghan R (2005). Association of chromosome 7, chromosome 10 and EGFR gene amplification in glioblastoma multiforme. Clin Neuropathol.

[49] Crespo I, Vital AL, Nieto AB, Rebelo O, Tao H, Lopes MC (2011). Detailed characterization of alterations of chromosomes 7, 9, and 10 in glioblastomas as assessed by single-nucleotide polymorphism arrays. J Mol Diagn.

[50] Sturm D, Bender S, Jones DT, Lichter P, Grill J, Becher O (2014). Paediatric and adult glioblastoma: multiform (epi)genomic culprits emerge. Nat Rev Cancer.

[51] Huhn SL, Mohapatra G, Bollen A, Lamborn K, Prados MD, Feuerstein BG (1999). Chromosomal abnormalities in glioblastoma multiforme by comparative genomic hybridization: correlation with radiation treatment outcome. Clin Cancer Res.

[52] Kuo KT, Guan B, Feng Y, Mao TL, Chen X, Jinawath N (2009). Analysis of DNA copy number alterations in ovarian serous tumors identifies new molecular genetic changes in low-grade and high-grade carcinomas. Cancer Res.

[53] Gusev Y, Riggins RB, Bhuvaneshwar K, Gauba R, Sheahan L, Clarke R (2013). In silico discovery of mitosis regulation networks associated with early distant metastases in estrogen receptor positive breast cancers. Cancer Informat.

[54] Clark K, Vendt B, Smith K, Freymann J, Kirby J, Koppel P (2013). The Cancer Imaging Archive (TCIA): maintaining and operating a public information repository. J Digit Imaging.

[55] Cerami E, Gao J, Dogrusoz U, Gross BE, Sumer SO, Aksoy BA (2012). The cBio cancer genomics portal: an open platform for exploring multidimensional cancer genomics data. Cancer Discov.

[56] Rhodes DR, Kalyana-Sundaram S, Mahavisno V, Varambally R, Yu J, Briggs BB (2007). Oncomine 3.0: genes, pathways, and networks in a collection of 18,000 cancer gene expression profiles. Neoplasia.

[57] Elgamal OA, Park JK, Gusev Y, Azevedo-Pouly AC, Jiang J, Roopra A (2013). Tumor suppressive function of mir-205 in breast cancer is linked to HMGB3 regulation. PLoS One.

[58] Lin L, Yao Z, Bhuvaneshwar K, Gusev Y, Kallakury B, Yang S (2014). Transcriptional regulation of STAT3 by SPTBN1 and SMAD3 in HCC through cAMP-response element-binding proteins ATF3 and CREB2. Carcinogenesis.

[59] Zhi X, Lin L, Yang S, Bhuvaneshwar K, Wang H, Gusev Y (2015). betaII-Spectrin (SPTBN1) suppresses progression of hepatocellular carcinoma and Wnt signaling by regulation of Wnt inhibitor kallistatin. Hepatology.

[60] Feizi A, Bordel S (2013). Metabolic and protein interaction sub-networks controlling the proliferation rate of cancer cells and their impact on patient survival. Sci Rep.

[61] Kolassa JE, Seifu Y (2013). Nonparametric multivariate inference on shift parameters. Acad Radiol.

[62] BCHB-594 Translational Bioinformatics. [http://courses.georgetown.edu/index.cfm?Action=View&CourseID=BCHB-594&AcademicYear=2013&AcademicTerm=FallSpring] Accessed: 9 Feb 2015.

[63] Athey BD, Braxenthaler M, Haas M, Guo Y (2013). tranSMART: an open source and community-driven informatics and data sharing platform for clinical and translational research. AMIA Jt Summits Transl Sci Proc.

[64] Canuel V, Rance B, Avillach P, Degoulet P, Burgun A (2015). Translational research platforms integrating clinical and omics data: a review of publicly available solutions. Brief Bioinform.

[65] Muirhead LJ (2012). Surgical systems biology and personalized longitudinal phenotyping in critical care. Pers Med.

[66] Chen X, Sun X, Hoshida Y (2014). Survival analysis tools in genomics research. Hum Genomics.

