# Supplementary figures and images for: G-DOC Plus – an integrative bioinformatics platform for precision medicine
Source: BMC Bioinformatics. 2016 Apr 30;17:193. doi: 10.1186/s12859-016-1010-0 (PMC4851789; doi:10.1186/s12859-016-1010-0)

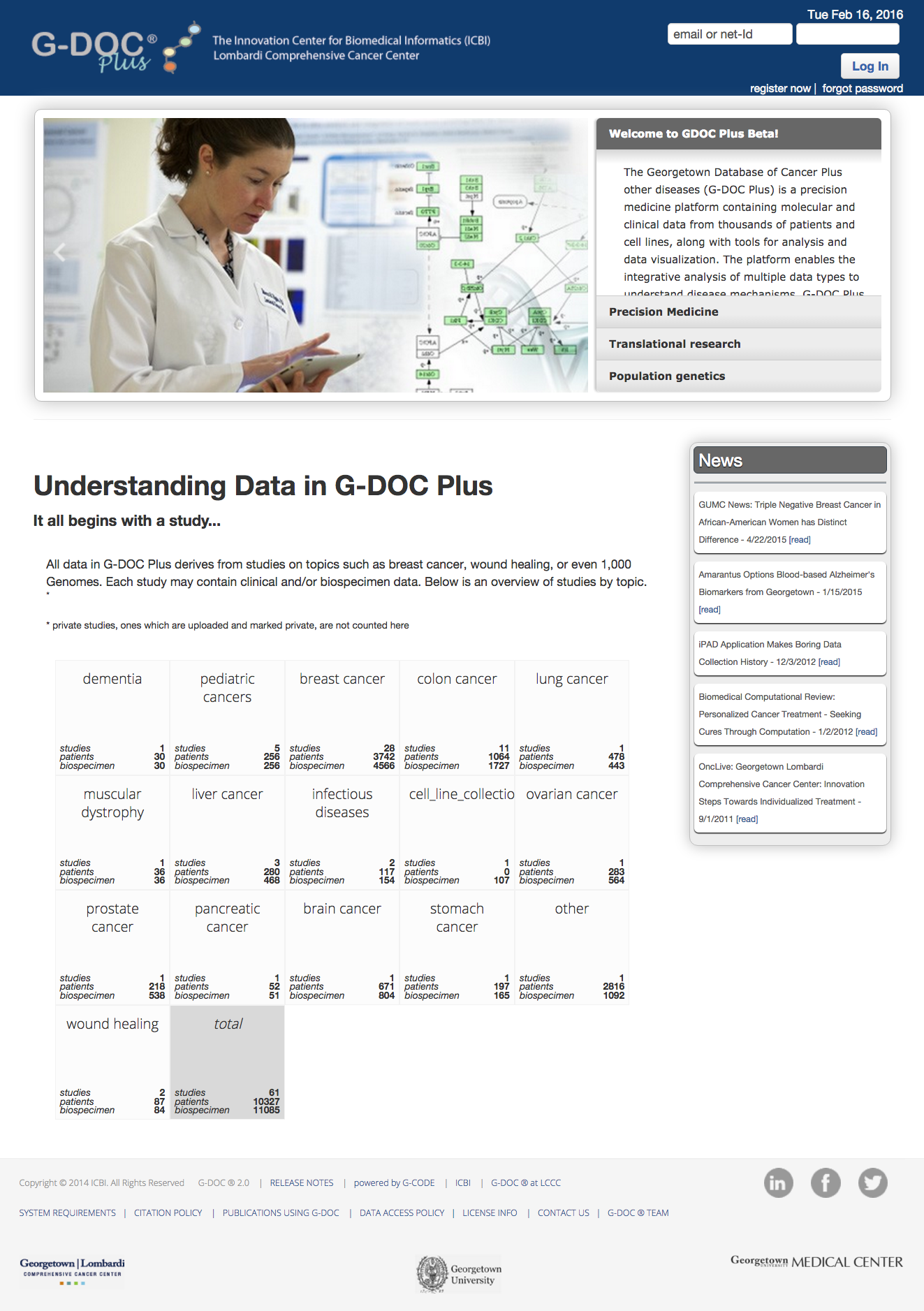

Supplement: Additional file 1: — Front page of G-DOC Plus showing total number of studies, and samples in various disease types. (PNG 885 kb) [file 12859_2016_1010_MOESM1_ESM.png]

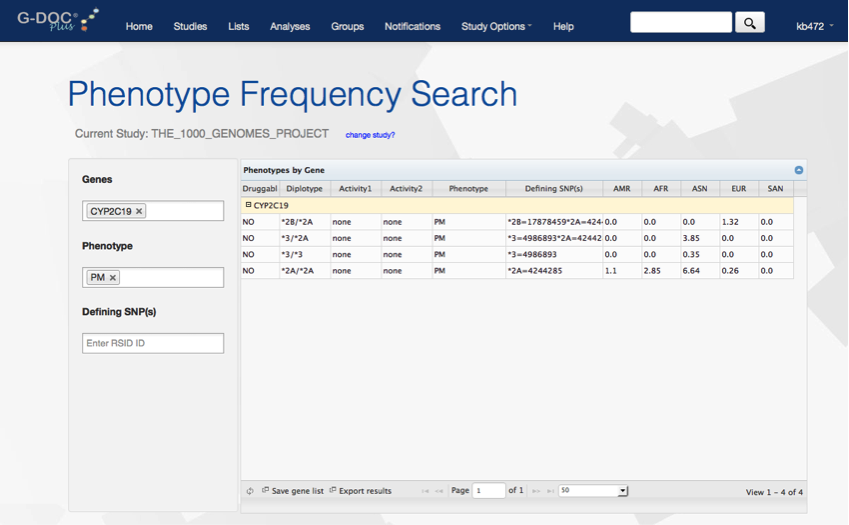

Supplement: Additional file 3: — Screen shot of 1000 genomes use case with settings for poor metabolizers. (PNG 133 kb) [file 12859_2016_1010_MOESM3_ESM.png]

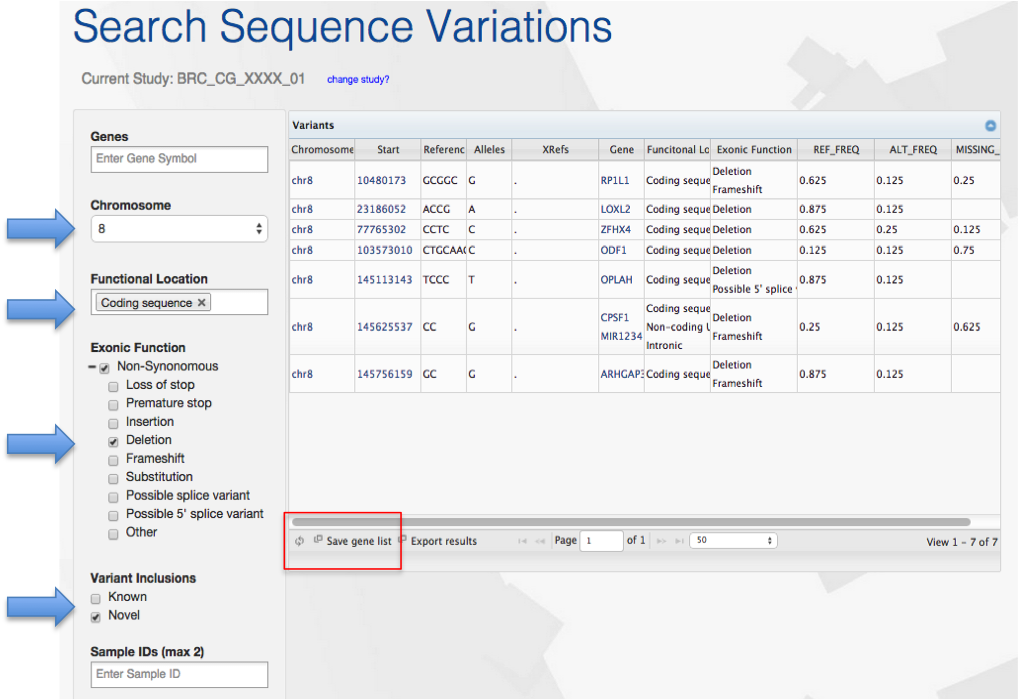

Supplement: Additional file 4: — Screen shot of variant search use case with the settings. (PNG 265 kb) [file 12859_2016_1010_MOESM4_ESM.png]

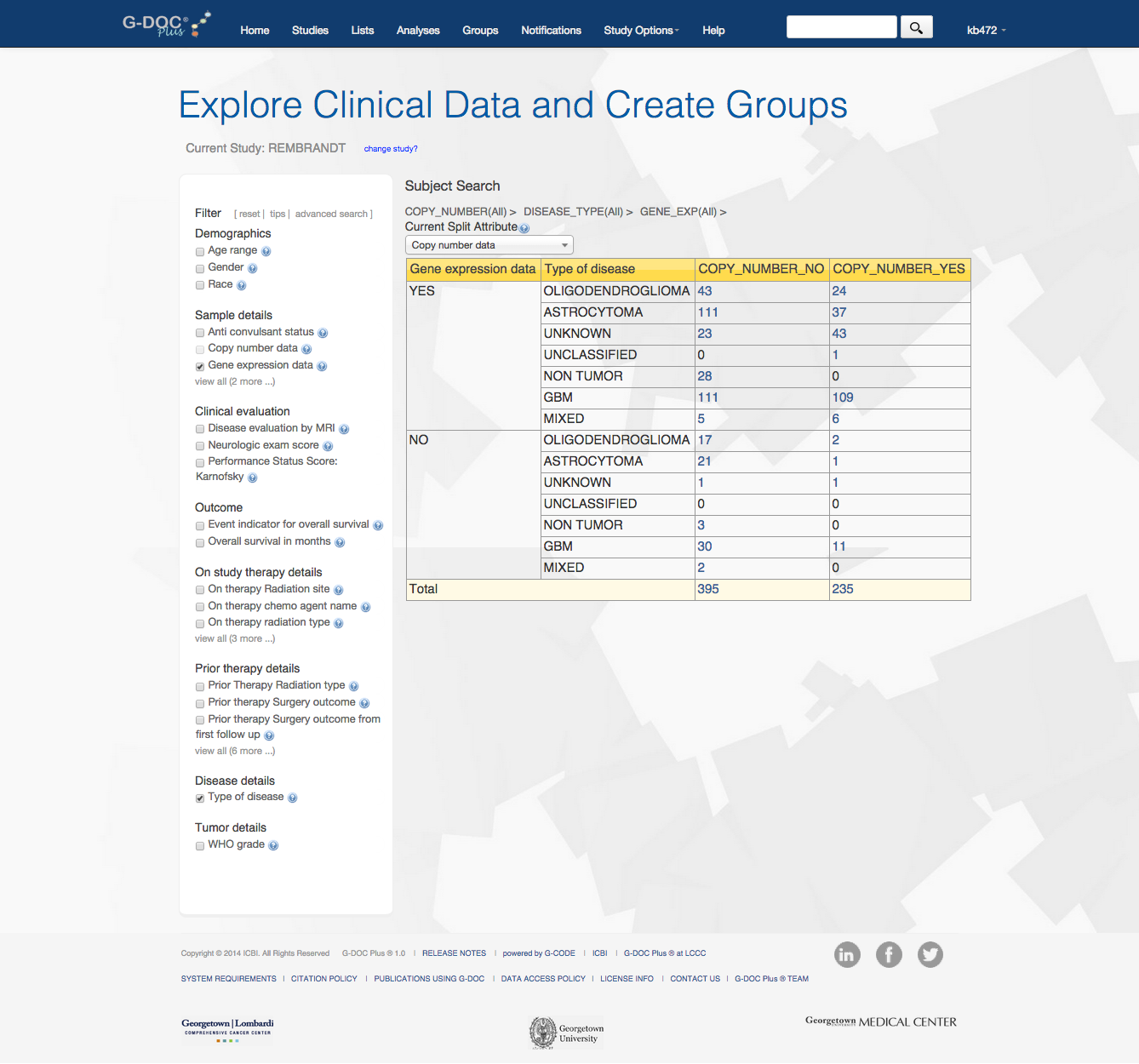

Supplement: Additional file 5: — Screen shot of G-DOC Plus showing clinical cohort creation. (PNG 424 kb) [file 12859_2016_1010_MOESM5_ESM.png]

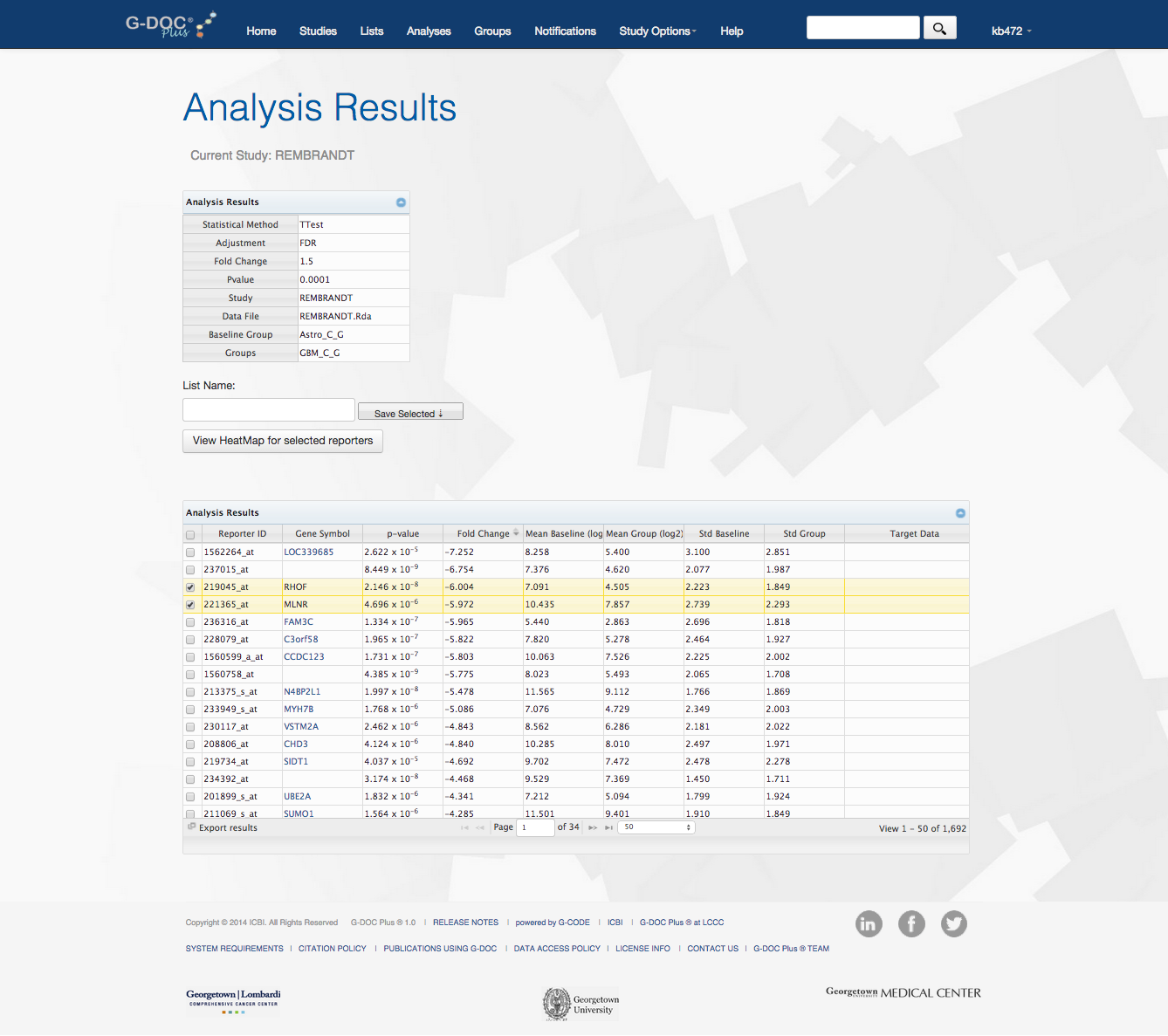

Supplement: Additional file 6: — T-test with FDR performed on gene expression data Astrocytoma and GBM patients. Screen shot of results on G-DOC Plus to show DEGs. (PNG 344 kb) [file 12859_2016_1010_MOESM6_ESM.png]

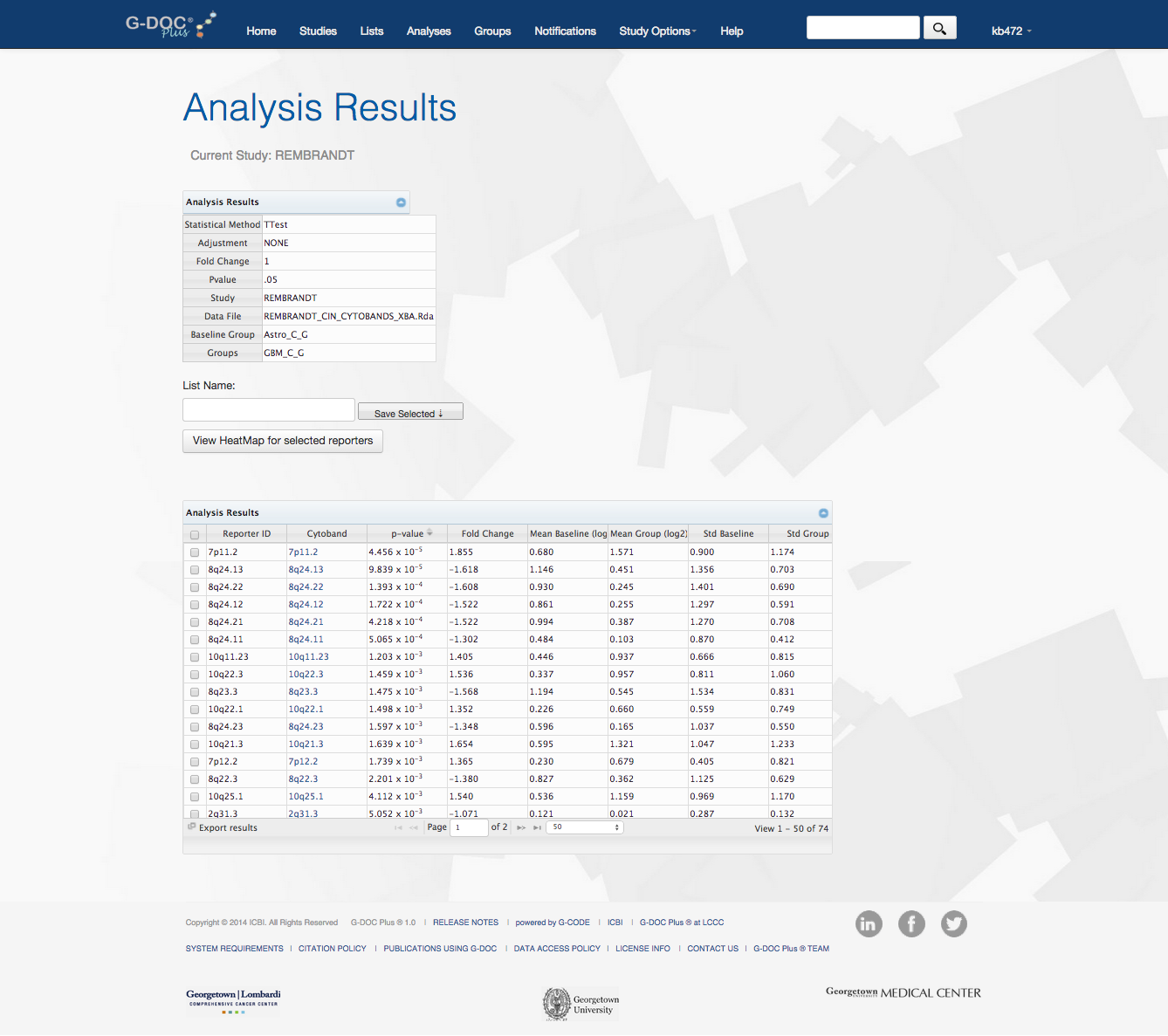

Supplement: Additional file 7: — Rembrandt T-test with CIN cytobands. (PNG 344 kb) [file 12859_2016_1010_MOESM7_ESM.png]
