# Supplementary material for: G-DOC Plus – an integrative bioinformatics platform for precision medicine
Source: BMC Bioinformatics. 2016 Apr 30;17:193. doi: 10.1186/s12859-016-1010-0 (PMC4851789; doi:10.1186/s12859-016-1010-0)
Supplement: Additional file 2: — What’s new in G-DOC Plus? (DOCX 87 kb) [file 12859_2016_1010_MOESM2_ESM.docx]

## What’s new in G-DOC *Plus*?

| **DISEASE TYPES** | **Cancer / Non-cancer** | **G-DOC** | **G-DOC *Plus*** |
| --- | --- | --- | --- |
| Breast cancer | Cancer | Yes | Yes |
| Colon cancer | Cancer | Yes | Yes |
| Liver cancer | Cancer | Yes | Yes |
| Pancreatic cancer | Cancer | Yes | Yes |
| Prostrate cancer | Cancer | Yes | Yes |
| Ovarian cancer | Cancer | Yes | Yes |
| Stomach cancer | Cancer | Yes | Yes |
| Pediatric cancer | Cancer | No | Yes |
| Dementia | Non-cancer | No | Yes |
| Duchene Muscular Dystrophy (DMD) | Non-cancer | No | Yes |
| Wound healing | Non-cancer | No | Yes |
| NCI-60 cell line collection | Cancer | No | Yes |
| The 1000 genomes study | Non-cancer | No | Yes |
| Brain cancer | Cancer | No | Yes |
| Infectious diseases | Non-cancer | No | Yes |
| Lung Cancer | Cancer | No | Yes |

| **DATA TYPES** | **G-DOC** | **G-DOC *Plus*** |
| --- | --- | --- |
| mRNA | Yes | Yes |
| microRNA | Yes | Yes |
| Metabolomics | Yes | Yes |
| Copy Number | Yes | Yes |
| Medical images | No | Yes |
| WGS | No | Yes |

|  | **G-DOC *Plus*** |
| --- | --- |
| Back end architecture | - Backend architecture re-designed into a modular structure (Grails plugin architecture) allowing for a flexible and extendible framework for new analysis modules to be easily added to the platform.  - WGS and Medical images stored in the cloud. |
| Web interface | Re-designed for better end user experience |
